# Detection of Shiga toxin-producing *Escherichia coli* (STEC) O157:H7, O26, O45, O103, O111, O121, and O145, and *Salmonella* in retail raw ground beef using the DuPont™ BAX® system

**DOI:** 10.3389/fcimb.2014.00081

**Published:** 2014-06-18

**Authors:** Jamie L. Wasilenko, Pina M. Fratamico, Christopher Sommers, Daniel R. DeMarco, Stephen Varkey, Kyle Rhoden, George Tice

**Affiliations:** ^1^United States Department of Agriculture, Agricultural Research Service, Eastern Regional Research CenterWyndmoor, PA, USA; ^2^United States Department of Agriculture, Agricultural Research Service, Richard Russell Research CenterAthens, GA, USA; ^3^DuPont Nutrition and Health, DuPont Experimental StationWilmington, DE, USA

**Keywords:** Shiga toxin-producing *E. coli*, *Salmonella*, ground beef, detection, PCR, O157:H7, non-O157 STEC

## Abstract

Shiga toxin-producing *Escherichia coli* (STEC) and *Salmonella* are food-borne pathogens commonly associated with beef, and reliable methods are needed to determine their prevalence in beef and to ensure food safety. Retail ground beef was tested for the presence of *E. coli* O157:H7, STEC serogroups O26, O45, O103, O111, O121, and O145, and *Salmonella* using the DuPont™ BAX® system method. Ground beef (325 g) samples were enriched in 1.5 L of TSB with 2 mg/L novobiocin at 42°C for 18 h, and then evaluated using the BAX® System real-time PCR assays for *E. coli* O157:H7 and STEC suite, and the BAX® System standard PCR assays for *E. coli* O157:H7 MP and *Salmonella*. Samples positive for STEC target genes by the BAX® System assays were subjected to immunomagnetic separation (IMS) and plating onto modified Rainbow Agar O157. Enrichments that were PCR positive for *Salmonella* were inoculated into RV broth, incubated for 18 h at 42°C, and then plated onto XLT-4 agar. Presumptive positive STEC and *Salmonella* colonies were confirmed using the BAX® System assays. Results of the BAX® System STEC assays showed 20/308 (6.5%) of samples positive for both the Shiga toxin (*stx*) and intimin (*eae*) genes; 4 (1.3%) for *stx, eae*, and O26; 1 (0.3%) for *stx, eae*, and O45; 3 (1%) for *stx, eae*, and O103; and 1 (0.3%) for *stx, eae*, and O145. There were also 3 samples positive for *stx, eae*, and more than one STEC serogroup. Three (1.0%) of the samples were positive using the BAX® System real-time *E. coli* O157:H7 assay, and 28 (9.1%) were positive using the BAX® System *Salmonella* assay. STEC O103 and *E. coli* O157:H7 were isolated from 2/6 and 2/3 PCR positive samples, respectively. *Salmonella* isolates were recovered and confirmed from 27 of the 28 *Salmonella* PCR positive samples, and a portion of the isolates were serotyped and antibiotic resistance profiles determined. Results demonstrate that the BAX® System assays are effective for detecting STEC and *Salmonella* in beef.

## Introduction

The USDA Food Safety and Inspection Service (FSIS) classified *Escherichia coli* O157:H7 as an adulterant in raw ground beef and began a verification testing program for this pathogen in 1994 in response to a large outbreak associated with undercooked ground beef. More recently, it has become evident that non-O157 Shiga toxin-producing *E. coli* (STEC), particularly STEC serogroups O26, O45, O103, O111, O121, and O145 (referred to as the top six non-O157 STEC) cause illnesses similar to those caused by *E. coli* O157:H7 (Gould et al., 2013). Cattle are a major reservoir for STEC in the U.S., and outbreaks due to non-O157 STEC, including serogroups O26 and O111 have been associated with beef or contact with cattle (Paton et al., 1996; Ethelberg et al., 2009; Kaspar et al., 2010). Thus, the FSIS declared the top six non-O157 STEC as adulterants in beef trim, and FSIS verification testing for these pathogens began in June 2012 in domestic and imported beef manufacturing trimmings (Anonymous, 2011).

Cattle are also a reservoir for *Salmonella*, and ground beef has been implicated in a number of food-borne outbreaks of salmonellosis (Talbot et al., 2006). Various methods have been described for detection of *Salmonella*, and pre-enrichment can be performed in media such as Universal Enrichment Broth, lactose broth, trypticase soy broth, buffered peptone water, nutrient broth and others, with or without selective agents, followed by selective enrichment (Andrews et al., 2011; Anonymous, 2013a). A number of enrichment media have also been described for detection of *E. coli* O157:H7 and the non-O157 STEC (Wang et al., 2013). The methods in the FSIS Microbiology Laboratory Guidebook for detection of non-O157 STEC and O157:H7 involved enrichment in modified TSB (mTSB) containing 8 mg/L of novobiocin (Anonymous, 2012a,b), and in the current methods, mTSB with no novobiocin in used for enrichment (Anonymous, 2013c,d). Screening to eliminate negative samples can by performed by PCR-based assays, and this is followed by immunomagnetic separation (IMS) to concentrate and separate the target pathogens, plating onto selective and differential agars, and confirmation of presumptive positive colonies. The ISO/TS 13136:2012 method employs a similar approach but only targets 5 of the top STEC (O157, O111, O26, O103, and O145) (ISO, 2012).

Since STEC and *Salmonella* can contaminate beef, it would be practical to detect the pathogens from the same beef sample, and the ability to use a common enrichment medium for *E. coli* O157:H7, the top six non-O157 STEC, and *Salmonella* would result in savings in cost and time compared to testing for the pathogens separately. The objective of this study was to conduct a survey of retail ground beef using the BAX® system PCR assays for screening to determine the presence of *E. coli* O157:H7, the top six non-O157 STEC, and *Salmonella*. Isolation methods were used to recover the pathogens from screen positive beef enrichments, and the *Salmonella* strains were characterized to determine their serotypes and antibiotic resistance profiles.

## Materials and methods

### Beef sample enrichments

Aerobic plate counts (APC) were performed periodically on the retail ground beef samples (*n* = 29) by combining 65-g portions with 585 ml of buffered peptone water (BPW), making serial dilutions, plating onto Aerobic Count Petrifilms (3M, St. Paul, MN) that were incubated at 35°C overnight, and colonies were enumerated. APCs per gram of beef were calculated. For testing using the BAX® System PCR assays, 325 g of ground beef (8–27% fat), purchased from retail grocery stores in Pennsylvania and Georgia during the period of June to October 2012, were homogenized with 1.5 L of pre-warmed (42°C) tryptic soy broth (TSB) (BD Biosciences, Sparks, MD) (17 g pancreatic digest of casein, 5 g sodium chloride, 2.5 g dipotassium phosphate, 3 g papaic digest of soybean meal, 2.5 g dextrose) with 2 mg/L novobiocin (Sigma-Aldrich, St. Louis, MO) in Whirlpack filter bags (Nasco, Fort Atkinson, WI), mixed in a Stomacher (Seward Laboratory Systems, Inc., Bohemia, NY) for 1 min, and then incubated at 42°C for 18 h before testing with the BAX® System assays.

### BAX® system assays

The BAX® System PCR assays used in this study were the following: BAX® System real-time PCR assay suite for STEC-Screening (*stx* and *eae*), Panel 1 *E. coli* (O26, O111, and O121), Panel 2 *E. coli* (O45, O103, and O145), and the BAX® System Real-Time PCR assay for *E. coli* O157:H7. The BAX® System PCR assay for *E. coli* O157:H7 MP and the BAX® System PCR assay for *Salmonella* were also used (DuPont Nutrition and Health, Wilmington, DE).

### Testing of ground beef enrichments

For *E. coli* O157:H7 and STEC testing, 20 μl of enrichment were added to 200 μl of prepared BAX® System lysis reagent in cluster tubes. For *Salmonella* testing, 5 μl of enrichment were added to the lysis reagent. Lysis was performed by heating the tubes for 20 min at 37°C and 10 min at 95°C, and then cooling tubes at 4°C for at least 5 min. For the BAX® System real-time PCR assays (STEC Screening, STEC Panel 1, STEC Panel 2, and *E. coli* O157:H7), 30 μl of lysate were used to hydrate tablets in PCR tubes. PCR tubes were loaded into the BAX® System Q7 instrument, and a full process was run according to the procedure described in the BAX® System User Guide and analyzed using software version 3.2. For standard assays (*E. coli* O157:H7 MP and *Salmonella*), 50 μl of lysate were used to hydrate tablets in PCR tubes. PCR tubes were loaded into the BAX® System Classic instrument, and a full process was run according to the procedure described in the BAX® System User Guide. For the *E. coli* O157:H7 MP assay, the MP Express program was used.

### IMS, plating, and confirmation of STEC

Enrichments that tested positive for *stx, eae* and an O-group or O157:H7 underwent IMS. Enrichments were filtered using a sterile 40 μm cell strainer, and then 1 mL of the filtered enrichment was added to SDIX (Newark, DE) RapidChek O-specific immunomagnetic capture beads (50 μl) and O157 Invitrogen Dynabeads EPEC/VTEC (Invitrogen, Grand Island, NY) (20 μl) immunomagnetic beads. Enrichments and immunomagnetic beads were mixed by rotation at room temperature for 15 min, and then IMS on a Dynal MPC-S (Invitrogen) platform was performed. Enrichment-magnetic bead mixtures were exposed to the magnet for 5 min, and then the liquid was removed, and 1 ml of E-buffer Buffered Peptone Water (100 ml), Tween-20 (50 μl), and bovine serum albumin (0.5 g) was used to wash the beads. The washes were repeated 2 more times, and the beads were resuspended in 1 ml of E-buffer.

An aliquot of each bead suspension was spread onto modified Rainbow Agar O157 (mRBA) plates according to USDA FSIS MLG Chapter 5B.03 (3) (Rainbow Agar O157 [Biolog, Hayward, CA] containing 5 mg/l sodium novobiocin, 0.05 mg/l cefixime, 0.15 mg/l potassium tellurite). The undiluted bead suspension, as well as 1:10 and 1:100 dilutions were plated onto mRBA plates. An additional aliquot was subjected to an acid treatment, adding 25 μl of 1 N HCl acid into 450 μl of the bead suspension, rotating for 1 h before neutralizing with 475 μl of E-buffer. Bead suspensions were plated onto mRBA plates both undiluted and a 1:10 dilution from the acid treated suspensions. All plates were incubated at 35°C for 20–24 h. Presumptive positive colonies were tested by latex agglutination (Medina et al., 2012) using latex beads for non-O157 STEC obtained from Abraxis LLC (Warminster, PA) and for *E. coli* O157:H7 (Thermo Scientific, Waltam, MA), and then confirmed using the BAX® System Real-Time PCR assay for *E. coli* O157:H7 or the BAX® System Real-Time PCR STEC suite. Colonies for confirmation were resuspended in 1 ml of sterile water, and 5 μl were added to BAX® cluster tubes containing 200 μl of prepared BAX® System lysis buffer. Lysis was performed by heating the tubes for 20 min at 37°C and 10 min at 95°C, and then cooling tubes at 4°C for at least 5 min.

### Isolation and determination of serotype and antibiotic resistance profiles of *Salmonella*

Enrichments (0.1 ml) that tested positive for *Salmonella* using the BAX® System *Salmonella* assay were inoculated into 10 ml of Rappaport-Vassiliadis broth (RV) (EMD Chemicals Inc., Gibbstown, NJ) and incubated for 18 h at 42°C, and then the cultures were plated onto XLT-4 agar plates (BD Biosciences). Plates were incubated at 37°C for 18 h before confirming colonies. Colonies for confirmation were resuspended in 1 ml of sterile water, and 5 μl of the dilutions were added to cluster tubes containing 200 μl of BAX® System lysis buffer, and then lysis was performed as described above. The BAX® System PCR assay for *Salmonella* was used to confirm the lysates. The *Salmonella* isolates were serotyped, and the antibiotic resistance profiles were determined at the USDA National Veterinary Services Laboratory in Ames Iowa, against ampicillin, azithromycin, cefoxitin, ceftiofur, ceftriaxone, chloramphenicol, ciprofloxacin, gentamicin, kanamycin, nalidixic acid, streptomycin, sulfisoxazole, tetracycline, and trimethoprim/sulphamethoxazole, which were selected based on *Salmonella* isolated from cattle.

## Results and discussion

Retail ground beef (*n* = 308) was obtained from grocery stores in Pennsylvania and Georgia and tested for the presence of *E. coli* O157:H7, the top-6 non-O157 STEC serogroups (O26, O45, O103, O111, O121, and O145), and *Salmonella* using commercial BAX® System assays. Portions of random samples (*n* = 29) each week were analyzed for aerobic bacteria, and aerobic plate counts ranged from 10^1.31^ to 10^6.78^ CFU/g, with an average of 10^5.66^ CFU/g from the 29 samples analyzed. The 308 ground beef samples were subjected to enrichment in TSB containing 2 mg/L of novobiocin. After testing with the BAX® System PCR assays targeting STEC and *Salmonella*, an aliquot of the samples positive for *Salmonella* was removed and subjected to a second enrichment in RV broth prior to plating onto XLT-4 agar. *E. coli* O157:H7 and other STEC serogroups, as well as *Salmonella* can be found as contaminants in beef products, and thus use of a common enrichment/pre-enrichment medium that is suitable for growth of all of these pathogens simplifies screening since the pathogens can then be detected from the same sample. The method in the FSIS MLG uses the BAX kits for screening for non-O157 STEC and O157:H7 (Anonymous, 2013d,e), and samples that are positive using the BAX assays are then subjected to IMS, isolation, and confirmation. Thus, in the current study, only samples positive by the BAX kits were subjected to further testing to attempt to isolate the target pathogens. The BAX kits for non-O157 STEC and O157:H7 have recently been evaluated (Fratamico et al., 2014). The assays were highly specific for the STEC serogroups, and the sensitivity of assays for the different PCR targets was ≥ 1.23 × 10^3^ CFU/mL using pure cultures. It would be of interest to also compare the BAX system assays to the ISO/TS 13136:2012 method (ISO, 2012).

Fourteen percent (45/308) and 23% (72/308) of the retail ground beef samples tested were positive for *stx* and *eae* after enrichment, respectively (Tables 1, 2). Previous reports of ground beef samples demonstrated that 8.5% (in the Washington D.C. area) and 24.4% (across the U.S.) of retail samples tested positive for *stx* (Bosilevac and Koohmaraie, 2011; Ju et al., 2012), and the results of the current study fall within that range. A previous study by Bosilevac and Koohmaraie (2011) reported that 10.1% of retail ground beef samples were *stx* and *eae* positive, which is similar to the results of this study in which 6.5% (20/308) of samples were positive for the *stx* and *eae* genes. Of the 20 samples positive for both *stx* and *eae*, four were positive for O26, one for O45, three for O103, one for O145, two for both O26 and O103, and one for O26, O103, and O121 (Table 3). Recovery of the non-O157 STEC from enrichments that were positive for *stx* and *eae* was carried out according to the FSIS MLG 5B procedure (Anonymous, 2012a). STEC O103 isolates were recovered from 2 of the 6 *stx, eae*, and O103 positive samples. In the remaining samples potentially containing non-O157 STEC, isolates were not recovered from any of the enrichments positive for *stx, eae*, and at least one of the target O-groups. Only a portion of the samples that was positive for top-six STEC serogroup-specific genes were positive for *stx* and/or *eae*, and a number of these samples were positive for more than one non-O157 STEC serogroup gene. One possibility for the inability to recover STEC is that in some of the samples positive for the *stx, eae*, and the top six O-group-specific genes, they were not carried by the same cell/strain, and therefore, it was not possible to recover and confirm an STEC isolate. For example, there could have been non-STEC O26, O45, O103, O121, and O145 strains in the samples, as well as other *E. coli* strains carrying *stx* and/or *eae*. Indeed during this study, Shiga toxin-negative O26, O103, and O145 isolates were recovered after IMS even though the PCR screen was positive for *stx*. Five isolates were recovered from four different samples, consisting of one sample with an O26/*eae* positive isolate, one sample with an O103/*eae* positive isolate, another with an isolate positive for the O145 marker, and another had an O26/*eae* positive isolate, as well as an O103/*eae* positive isolate (data not shown). Furthermore, the non-O157 STEC are a very heterogeneous group of organisms, there are no suitable markers to aid in identification such as the inability to ferment sorbitol and lack of β-glucuronidase activity as in O157:H7, and they vary in terms of sensitivity to selective agents. Thus, these factors may have contributed to the inability to isolate the non-O157 STEC in some samples that were positive for *stx, eae*, and a top six O-group-specific gene. Although several new selective and differential agar media have been developed for isolating STEC, the identification of distinct markers and growth requirements that allow growth of all STEC serogroups (even different strains within a serogroup) and for differentiation of one serogroup from another and from non-pathogenic *E. coli* that can be exploited in media development has been challenging. Tzschoppe et al. (2012) showed that STEC strains that did not grow on CHROMagar STEC lacked *terB* (tellurite resistance), and others have also shown that growth on this agar correlates with resistance to tellurite (Hirvonen et al., 2012). Tillman et al. (2012) found that lowering the concentration of tellurite in the FSIS Rainbow Agar O157 (RBA) formulation to 0.15 mg/L allowed growth of some STEC O45, O103, O111, and O121 strains that did not grow on RBA with tellurite at 0.8 mg/L.

**Table 1:** **Detection of STEC and *Salmonella* in 308 retail beef samples using the BAX® System real-time assays and the number of samples in which a colony was confirmed**.

**Targets present**	**Total No. samples (% total)**	**Pathogen confirmed (No. of samples in which a colony was confirmed)**
none	150 (48.7%)	
*eae*	21 (6.8%)	
O45	18 (5.8%)	
*stx*	11 (3.6%)	
O103	10 (3.2%)	
*Salmonella*	10 (3.2%)	*Salmonella* (10)
O121	9 (2.9%)	
O103, *eae*	7 (2.3%)	
*stx, eae*	7 (2.3%)	
O26	6 (1.9%)	
O45, *eae*	5 (1.6%)	
O26, *eae*	4 (1.3%)	
O26, *stx, eae*	3 (1.0%)	
O45, *stx*	3 (1.0%)	
O45, O121	3 (1.0%)	
O103, *stx*	2 (0.6%)	
O103, *stx, eae, Salmonella*	2 (0.6%)	*Salmonella* (1), *Salmonella* and O103 (1)
O26, O121, *eae*	2 (0.6%)	
*eae, Salmonella*	2 (0.6%)	*Salmonella* (2)
O121, *stx*	2 (0.6%)	
O26, O45	2 (0.6%)	
O26, O103, *stx, eae*	2 (0.6%)	O103 (1)
O26, O103, *eae*	2 (0.6%)	
*stx, Salmonella*	2 (0.6%)	*Salmonella* (2)
O103, *Salmonella*	1 (0.3%)	*Salmonella* (1)
O103, *eae, Salmonella*	1 (0.3%)	*Salmonella* (1)
O103, *eae, Salmonella*, O157:H7 RT^*^	1 (0.3%)	
O103, *stx, eae*	1 (0.3%)	
O103, *stx, Salmonella*	1 (0.3%)	*Salmonella* (1)
O26, *stx*	1 (0.3%)	
O26, O103*, stx, Salmonella*	1 (0.3%)	*Salmonella* (1)
O45, *eae, Salmonella*	1 (0.3%)	*Salmonella* (1)
O121, *eae, Salmonella*	1 (0.3%)	*Salmonella* (1)
O45, O103, *stx*	1 (0.3%)	
O45, O103*, eae, Salmonella*	1 (0.3%)	*Salmonella* (1)
O45, *Salmonella*	1 (0.3%)	*Salmonella* (1)
O26, O103, O121, *eae*	1 (0.3%)	
O26, O103, O121, *eae, Salmonella*	1 (0.3%)	*Salmonella* (1)
O26, O103, O121, *stx, eae*	1 (0.3%)	
O26, O45, *eae*	1 (0.3%)	
O26, *stx, eae*, O157:H7 MP^*^	1 (0.3%)	
O157:H7 MP^*^, *Salmonella*	1 (0.3%)	*Salmonella* (1)
O26, O121, *stx*	1 (0.3%)	
*stx, eae*, O157:H7 RT^*^,O157:H7 MP^*^	1 (0.3%)	O157 (1)
O145, *stx, eae*, O157:H7 RT^*^, O157:H7 MP^*^	1 (0.3%)	O157 (1)
O45, *stx, eae*	1 (0.3%)	
O26, O103, *eae, Salmonella*	1 (0.3%)	*Salmonella* (1)

* *O157:H7 RT is a positive result using the BAX ® System real-time E. coli O157:H7 assay and O157:H7 MP is a positive result using the BAX ® System E. coli O157:H7 MP assay*.

**Table 2:** **Distribution of *stx* and *eae* virulence genes in 308 retail beef samples**.

**Target genes**	**No. (percentage) of PCR positive samples**
*stx*+^a^	45 (14.6%)
*eae*+	72 (23%)
*stx*+, *eae*+	20 (6.5%)
*stx*+, *eae*+, top 6 serogroup gene	12 (3.9%)
*stx*+, *eae*−^a^, top 6 serogroup gene	12 (3.9%)
*stx*−, *eae*+, O157^b^	1 (0.3%)
*stx*+, *eae*+, O157^b^	2 (0.6%)

(+) positive; (−) negative. ^*^The O157:H7 result was based on the BAX® System real-time E. coli O157:H7 assay.

**Table 3:** **PCR screen results for non-O157 STEC serogroup and virulence gene distribution in 308 ground beef samples analyzed**.

**Targets present**	**No. of samples (% total)**
*stx, eae*, and O26	4 (1.3%)
*stx, eae*, and O45	1 (0.3%)
*stx, eae*, and O103	3 (1%)
*stx, eae*, and O111	0 (0%)
*stx, eae*, and O121	0 (0%)
*stx, eae*, and O145	1 (0.3%)
*stx, eae*, O26, and O103	2 (0.6%)
*stx, eae*, O26, O103, and O121	1 (0.3%)

*E. coli* O157:H7 isolates were recovered from 2/3 of the samples positive by the BAX® System real-time *E. coli* assay (Table 1). The enrichments from which the two O157:H7 isolates were recovered were positive for the *stx* and *eae* genes, and these 2 enrichments were positive using the BAX® System *E. coli* O157:H7 MP assay (Table 2). The third BAX® System real-time *E. coli* assay positive sample was *eae* positive and *stx* negative using the BAX® System real-time PCR STEC suite, and an isolate was not recovered after IMS. It is worth noting here that the BAX® System real-time PCR assay for *E. coli* O157:H7 is a multiplex PCR test whereby the detection of two targets (labeled as “S” and “W”) is required for a positive result to be reported. Typically, when one organism contributes both targets (which has been demonstrated for *E. coli* O157:H7 through extensive inclusivity testing) (Burns et al., 2011), the respective cycle threshold (*Ct*) values should be relatively equivalent. In the case of this particular sample, the *Ct*-values for target “S” and “W” were approximately 12.4 cycles apart (30.6 for target “S” and 43 for target “W”). Upon visual inspection of the amplification plot for this result, a particularly strong amplification for target “S” was observed. On the contrary, the amplitude for target “W” was very low by comparison, and the Ct was calculated at the last possible cycle of the analysis. This, along with the negative BAX® System *E. coli* O157:H7 MP result and lack of *stx* target, suggests that targets “S” and “W” may have originated from two separate organisms. The BAX® System *E. coli* O157:H7 MP assay utilizes SYBR Green intercalation of PCR products whereas the BAX® System real-time PCR assay utilizes real-time amplification through Scorpion probe constructs. While there are some similarities between the two assays, the primer sequences, formulation, and data analysis are different, and this may account for differences observed between the assays.

In 2010, the per capita consumption of beef in the U.S. was 57.2 pounds (ca. 26,000 g) (http://www.explorebeef.org/CMDocs/ExploreBeef/Beef%20Market%20At%20a%20Glance%20FINAL8.3.12.pdf), which is equivalent to 80 325-g samples. If 14/308 samples were positive for *stx, eae*, and one or more of the top seven O-groups (Table 2), this represents 1/22 samples that were positive. Therefore, one would expect that 3.6 out of 80 325-g ground beef portions eaten by consumers per year would be positive for either the top six STEC or O157:H7. However, STEC were only recovered from 4 of the samples, and thus, it is not certain that all of the 14 samples actually contained one strain that was positive for *stx, eae*, and one of the target seven O-groups.

Out of the 308 ground beef samples tested, 28 (9.1%) were screen positive for *Salmonella* using the BAX® System assay. It has been reported that 4.2% of retail ground beef samples in the U.S were positive for *Salmonella* (Bosilevac et al., 2009), which is somewhat lower than the prevalence found in the current study. In retail meat samples collected in Turkey, however, 29.3, 21.3, and 16% of poultry meat, ground beef, and beef samples, respectively, were positive for *Salmonella*, and the most common serotype was *S*. Typhimurium (Arslan and Eyi, 2010). Of the 28 samples that were PCR positive for *Salmonella*, the pathogen was recovered and confirmed from XLT-4 agar from 27 of the samples (Table 1). *Salmonella* Oranienburg was isolated from one of the samples (#290) (Table 4) from which STEC O103 was isolated and confirmed, as well. The ability to grow and recover both *Salmonella* and non-O157 STEC from a single sample indicates that the single enrichment medium used here is effective for growth of both organisms. The *Salmonella* isolates recovered from 17 of the 28 PCR-positive samples were serotyped, and the antibiotic resistance profiles were determined. Results are shown in Table 4. The *Salmonella* serotypes included, *S*. Cerro, *S*. Anatum, *S*. Kentucky, *S*. Montevideo, *S*. Bovimorbificans, *S*. Newport, *S*. Muenchen, *S*. Schwarzengrund, *S*. Agona, and *S*. Oranienburg, with *S*. Montevideo being the most commonly identified (6/17 samples). The sample numbers from which the *Salmonella* strains were isolated are shown. From samples 176, 187, 211, 245, and 290, more than one colony was picked from the XLT-4 agar (for example 176.1 and 176.2, 187.1 and 187.2, etc.), and serotyping results showed that only one serotype was identified from each of these samples. The two *S*. Newport isolates recovered from sample 176 showed resistance to multiple classes of antibiotics (ampicillin, chloramphenicol, streptomycin, sulfisoxazole, and tetracycline) (Table 4). Previous studies on retail meat samples found *Salmonella* with resistance to ampicillin, streptomycin, and tetracycline to be a common pattern (Zhao et al., 2006). *S*. Newport has been listed as one of the top 10 most commonly identified serotypes in ground beef from 2000 to 2011 (Anonymous, 2013b), and the CDC lists *S*. Newport as one of the most commonly identified serotypes involved in human illness (CDC, 2011).

**Table 4:** **Salmonella serotypes isolated from the different ground beef samples and antibiotic resistance profiles of the isolates**.

**Sample number^a^**	***Salmonella* serotype**	**Resistance to antibiotics^b^**
83	*Salmonella* Cerro	
88	*Salmonella* Cerro	
93	*Salmonella* Anatum	
101	*Salmonella* Kentucky	
102	*Salmonella* Montevideo	
105	*Salmonella* Montevideo	
106	*Salmonella* Montevideo	
109	*Salmonella* Montevideo	
111	*Salmonella* Kentucky	
154	*Salmonella* Montevideo	
165	*Salmonella* Bovimorbificans	
176.1	*Salmonella* Newport	Ampicillin, chloramphenicol, streptomycin, sulfisoxazole, and tetracycline
176.2	*Salmonella* Newport	Ampicillin, chloramphenicol, streptomycin, sulfisoxazole, and tetracycline
187.1	*Salmonella* Muenchen	
187.2	*Salmonella* Muenchen	
211.1	*Salmonella* Schwarzengrund	
211.2	*Salmonella* Schwarzengrund	
236	*Salmonella* Agona	
245.1	*Salmonella* Montevideo	
245.2	*Salmonella* Montevideo	
290.1	*Salmonella* Oranienburg	
290.2	*Salmonella* Oranienburg	
290.3	*Salmonella* Oranienburg	

a *Sample numbers ending in “0.1, 0.2, or 0.3” refer to different colonies picked from the same XLT-4 agar plate*.

b *Isolates were tested for resistance to ampicillin, azithromycin, cefoxitin, ceftiofur, ceftriaxone, chloramphenicol, ciprofloxacin, gentamicin, kanamycin, nalidixic acid, streptomycin, sulfisoxazole, tetracycline, and trimethroprim/sulphamethoxazole. If no result is provided, then the isolates were susceptible to all of the antibiotics tested, or results were “No interpretation” for some of the antibiotics. No interpretation criteria were established by Clinical Laboratory Standards Institute for certain combinations of antimicrobial, animal species, and pathogen*.

## Conclusions

In summary, *E. coli* O157:H7, the top six non-O157 STEC serogroups, and *Salmonella* were detected in retail ground beef samples using the BAX® System real-time PCR assays, and the pathogens were isolated from a number of samples following enrichment. The use of mTSB with 2 mg/L of novobiocin may be a suitable medium for simultaneous enrichment of these pathogens from the same sample of ground beef; however, a second enrichment in RV broth and plating onto XLT-4 agar for samples that screen positive for *Salmonella* will facilitate isolation of *Salmonella* species. Finally, there is a need for improved selective and differential agar media that will enhance the ability to isolate non-O157 STEC.

### Conflict of interest statement

The authors declare that the research was conducted in the absence of any commercial or financial relationships that could be construed as a potential conflict of interest.

## Author note

Mention of trade names or commercial products is solely for the purpose of providing specific information and does not imply recommendation or endorsement by the U.S. Department of Agriculture.
